# Historical Retrospect

**Published:** 1826-01

**Authors:** 


THE LONDON

### Medical and Physical Journal.

Nº 1 OF VOL. LV.] JANUARY, 1826. [Nº 323.

For many fortunate discoveries in medicine, and for the detection of numerous errors, the world is indebted to the rapid circulation of Monthly Journals; and there never existed any work, to which the Faculty, in Europe and America, were under deeper obligations, than to the Medical and Physical Journal of London, now forming a long, but an invaluable, series.—RUSH.

### HISTORICAL RETROSPECT.

It has been customary with us, at the present season, to look back upon the path which had brought us to the close of another year, and to endeavour to call to mind and arrange the most prominent and interesting objects which had presented themselves to our notice. On sitting down for this purpose, and endeavouring to select some discoveries in medical science, or new applications of previous knowledge,—such as, by their value and importance, might justify us in separating them from the general mass,—we have felt more strongly than on any former occasion the difficulty of accomplishing this task in a satisfactory manner. Much has been done, when viewed in the aggregate. Many works have been written,—many essays published,—many cases recorded ; yet, when we come to examine them in the detail, we find little of pre-eminent merit, and what appeared to us most valuable we have given in various departments of former Numbers. To give the whole is impossible: to select where the demands are so numerous, and the claims so equal,—at best is difficult.

There are two objects which may be had in view in laying before our readers an Historical Retrospect: it may either be intended for general perusal, or be regarded merely as a work of reference on individual subjects.

To do any justice to this kind of compilation, unavoidably extends it so much as to interfere with (if not to supersede) some of the other departments; while, from its very nature, embracing as it does such a multiplicity of unconnected subjects, we strongly suspect that it was less read than other parts of the Journal, even when executed in the very superior manner which characterised the PROEMIA of our much-respected predecessor, Dr. HUTCHINSON. If, on the other hand, these Retrospective Essays be intended for reference only, then unquestionably they are not extended enough; nor would the limits of this Journal admit of their being so, were an entire Number to be devoted to each of them. No one, we presume, who was engaged in collecting information on any particular point of medical science, would content himself with the mere outline of which our Retrospect must necessarily consist. To these motives for discontinuing this department in its usual form, we may add another,—the delay which it presents to the publication of Original Communications, and the consequent disappointment to our Correspondents.

But, although we mean not to attempt in future to condense within a few pages the contents of many volumes, yet we purpose to give, at the beginning of each year, a list, or "Catalogue Raisonné," of all the most important Works, Papers, and Cases, published during the one which shall have just elapsed. By this plan, the other departments, with the exception of the INTELLIGENCE, will not be encroached upon; the general reader will find the usual matter for his perusal; and those interested in particular subjects will be directed to the best sources of information.—EDITORS.

